# Zinc-alpha2-glycoprotein in patients with acute and chronic kidney disease

**DOI:** 10.1186/1471-2369-14-145

**Published:** 2013-07-12

**Authors:** Inga Sörensen-Zender, Jan Beneke, Bernhard MW Schmidt, Jan Menne, Hermann Haller, Roland Schmitt

**Affiliations:** 1 Department of Nephrology, Hannover Medical School, Carl-Neuberg-Str. 1, Hannover D-30625, Germany

**Keywords:** Zinc-alpha2-glycoprotein, Hemodialysis, Acute kidney injury, Adipokine

## Abstract

**Background:**

Zinc-alpha2-glycoprotein (AZGP1) is a secreted protein which is synthesized in a variety of cell types. AZGP1 has functionally been implicated in lipid metabolism, the regulation of cell cycling and cancer progression. Previous studies have shown increased circulating AZGP1 levels in patients with chronic kidney disease but AZGP1 has not been investigated in acute kidney injury (AKI). In this study, serum AZGP1 levels were measured in acute and chronic kidney disease to test for a correlation to renal function and other clinical parameters.

**Methods:**

We performed ELISA based measurements of AZGP1 serum levels in 21 patients suffering from grade 3 AKI and in 20 chronic hemodialysis patients. In AKI patients, AZGP1 was first measured before initiation of acute renal replacement therapy and a second measurement was done during renal functional recovery. Sera of healthy blood donors served as controls. The association of AZGP1 with acute and chronic renal dysfunction was analysed, as well as the correlation with clinical parameters, body composition and biochemical variables.

**Results:**

Levels of circulating AZGP1 were significantly elevated in AKI patients. High initial levels of AZGP1 correlated with extra-renal complications but not with parameters of renal function. At follow-up, AZGP1 levels were still increased but now correlated significantly with creatinine, eGFR and urea. Circulating AZGP1 in chronic hemodialysis patients was higher than in AKI patients. An association to parameters of lipid metabolism was not found.

**Conclusions:**

This study illustrates that circulating AZGP1 is not only elevated in chronic hemodialysis patients but also sharply increases during the early phase of AKI. The unexpected association with extra-renal complications during AKI needs further exploration as it might point to unknown biological effects of AZGP1.

## Background

Zinc-alpha2-glycoprotein (AZGP1) is a secreted soluble protein which is synthesized in adipocytes and a variety of other cell types [1]. Although the definite role of AZGP1 remains unclear, it has been implicated in several biological processes. Besides its potential role in regulation of cell cycling and cancer progression [1-3], AZGP1 has been characterized as a functional modifier of lipid metabolism [4,5]. *In vitro*, AZGP1 stimulated lipolysis in adipocytes via activation of α3-adrenoceptors [6]. Injections into rats triggered expression of lipolytic enzymes, resulting in increased lipid mobilization and loss of body fat [7]. In mice with cancer cachexia the expression of AZGP1 was up-regulated suggesting that AZGP1 might act as a cancer cachexia factor [5]. Genetic deletion of AZGP1 resulted in increased body weight and reduced lipolytic capacity [8].

In 2008 an ELISA was developed which allowed measurement of AZGP1 levels in patient serum [9]. Several studies have since been conducted determining serum AZGP1 in different patient populations. While Stejskal et al. did not find significantly altered levels of AZGP1 in patients with metabolic syndrome [9], Yeung et al. showed a positive correlation between serum AZGP1 and BMI, insulin resistance and serum triglycerides [10]. Other conditions associated with increased serum AZGP1 levels included preeclampsia [11] and chronic heart failure [12], whereas decreased levels of AZGP1 were found in HIV patients [13]. Although some of these studies argued for an involvement of AZGP1 in changes of lipid metabolism, a clear cause-effect relationship has not been shown. Dysregulated lipid metabolism is thought to contribute importantly to the development of insulin resistance and increased mortality in patients with chronic kidney disease (CKD) [14]. Two recent studies explored serum AZGP1 levels in chronic hemodialysis (HD) patients and found significantly increased mean values [15,16]. Philipp et al. suggested that this increase was most likely caused by a loss of normal renal clearance of the protein [16]. While they could not find a correlation between AZGP1 levels and parameters of lipid metabolism or body composition [16], the second study showed an inverse correlation between circulating AZGP1 and adiposity [17]. So far, AZGP1 has not been investigated in the context of acute kidney injury (AKI).

In the present study we measured circulating AZGP1 in patients with AKI and in chronic HD patients to further explore AZGP1 in renal dysfunction.

## Methods

### Patients

We studied 20 adult patients on maintenance HD and 21 adult AKI patients admitted to Hannover Medical School during the EHEC serotype O104:H4 outbreak in 2011 [18]. Preexisting disease was systematically recorded and patients were classified into having comorbidities if diabetes, hypertension or coronary heart disease were present. Only patients with documented normal baseline renal function were included. All evaluated patients suffered from grade 3 AKI, according to the Acute Kidney Injury Network (AKIN) classification and required transient renal replacement therapy. All patients consented in writing to donating blood for scientific evaluation. Serum from 20 healthy subjects served as control and was obtained from blood donors at the Institute of Transfusion Medicine, Hannover Medical School. The protocol was carried out according to the Declaration of Helsinki and approved by the Institutional Review Board at Hannover Medical School. Standard laboratory values were measured at the central clinical laboratory.

### Quantification of circulating AZGP1 and other biochemical variables

Blood samples were obtained after an overnight-fast before HD in chronic HD patients. In AKI patients, blood was drawn before initiation of the first HD session as baseline value and follow-up samples were gathered 85 days in average after the last dialysis session. Serum AZGP1 was measured by a commercial enzyme-linked immunosorbent assay (Biovendor, Modrice, Czech Republic), according to the manufacturer’s instructions by investigators blinded to patients’ data. All measurements were performed in duplicate. The assay sensitivity was 0.673 ng/ml. The intraassay coefficient of variation was less than 5%. Serum creatinine, urea, cholesterol, triglycerides, CRP, leukocytes, AST, ALT and LDH were measured by standard laboratory methods in a certified laboratory.

### Clinical data collection

Age, gender and BMI were recorded in all patients and additional data were retrieved from patient charts. BMI was calculated with pre-dialysis body weight. Total body fat in maintenance HD patients was measured using standard multi-frequency bioelectrical impedance analysis at 30 minutes after a midweek dialysis session as previously described [19]. For AKI patients, data were retrieved from the centralized STEC-HUS consortium database which has previously been described [18]. eGFR was calculated using the CKD-EPI equation [20].

### Statistical analysis

Continuous variables are presented as median (interquartile range). Nonparametric ANOVA (Kruskall-Wallis test) was performed if more than 2 groups were analyzed. In this case Mann–Whitney U test was performed as posthoc test with a p-value < 0.025 as significance level. Otherwise data were compared using Mann–Whitney U or Wilcoxon test employing Spearman tests as appropriate. P-values <0.05 were considered significant. Stepwise linear regression analysis with forward selection was performed to identify parameters independently predicting AZGP levels at follow-up.

## Results

**Figure 1:**
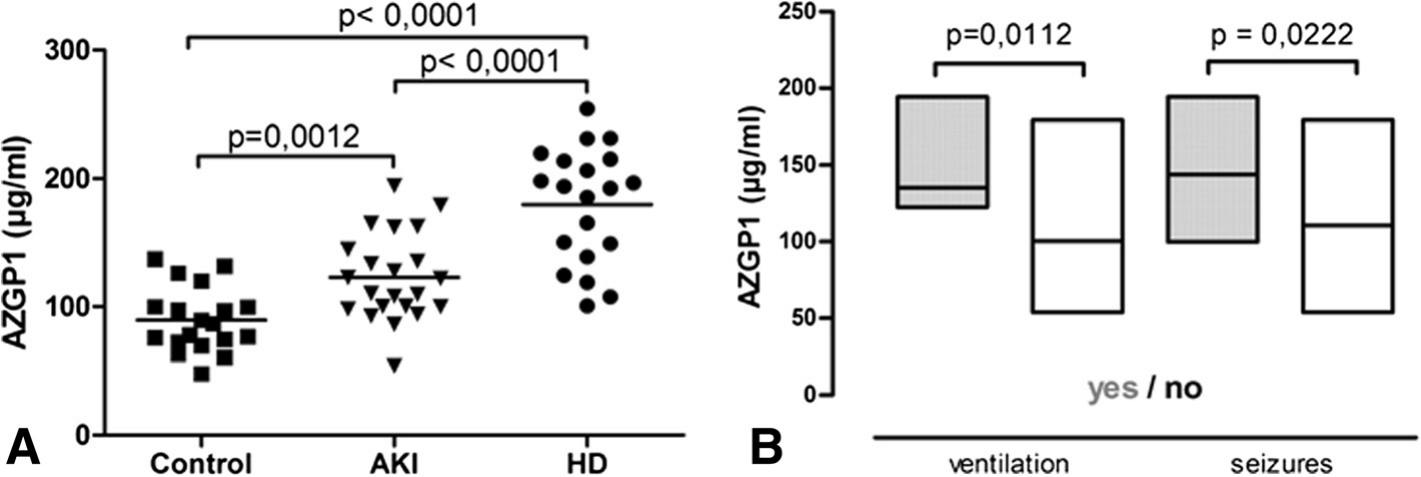
**AZGP1 serum levels are elevated in acute and chronic renal failure and correlate with extra-renal complications during AKI. A**: Mean serum AZGP1 serum levels were significantly higher in the AKI cohort than in controls at the time before the first session of dialysis but lower than in chronic HD patients. **B**: AZGP1 levels at admission significantly differed in patients who later would or would not require ventilation or developed seizures due to STEC-HUS induced cerebral changes.

All AKI patients included in our study were hospitalized for Shiga-toxin induced haemolytic uraemic syndrome (STEC-HUS) during the german outbreak 2011. AZGP1 was significantly higher in AKI patients than in control subjects (Figure 1A). In control subjects we found no correlation of AZGP1 to creatinine (82.3 (66.95 – 94.8) μmol/l) or eGFR (77.8 (66.9 – 91.3) ml/min). Similarly, among AKI patients, we found no significant correlation of initial AZGP1 to creatinine, urea and eGFR (Table 1). Initial AZGP1 levels higher or equal to median (121.87 μg/ml) positively correlated with age, pre-existing comorbidities and a more severe outcome of STEC-HUS (Table 2). All patients requiring ventilation belonged to this subgroup and patients of this group developed more often neurological symptoms including seizures and pathological EEGs (Table 2, Figure 1B).

**Table 1:** Univariate correlation between serum AZGP1 and other variables

	**AKI**		**AKI follow-up**	
	**n = 21**		**n = 21**	
	**Median (quantiles)**	**Spearmans’ coefficient; p-value**	**Median (quantiles)**	**Spearmans’ coefficient; p-value**
**creatinine** (μmol/l)	290 (218–409)	r = 0.093; p = 0.696	101 (79–132)	r = 0.776; p = <0.001
**eGFR** (ml/min)	16 (10.5 - 30)	r = −0.176; p = 0.458	52 (44–81)	r = −0.777; p = <0.001
**urea** (mmol/l)	15.8 (1.9 - 28.5)	r = 0.144; p = 0.546	7.6 (5.3 - 9.9)	r = 0.659; p = 0.002
**LDH** (U/l)	1311 (871–1791)	r = −0.168; p = 0.478	192 (171–237)	r = 0.887; p = < 0.0001
**CRP** (mg/l)	40.3 (21.8 - 101.8)	r = −0.104; p = 0.663	1 (1–6.5)	r = 0.62; p = 0.048
**AST** (U/l)	68 (58–89)	r = −0.139; p = 0.621	23 (20–26.5)	r = 0.49; p = 0.064
**ALT** (U/l)	32.5 (23–47.5)	r = −0.075; p = 0.775	17 (12–23.5)	r = 0.67; p = 0.006
**leukocytes** (10^3^/μl)	14.8 (8.8 - 19.6)	r = 0.390; p = 0.089	5.6 (4.8 - 6.7)	r = 0.455; p = 0.044

**Table 2:** Difference between AKI patients with AZGP1 higher/lower than median

**Admission AZGP1**	**≥ median**	**< median**	**p-value**
	**n = 11**	**n = 10**	
**basic parameters**			
**age (years)**	54 (48–70)	37 (23–46)	0.039
**comorbidities, n (%)** hypertension, diabetes mellitus, coronary heart disease	7 (63.6)	1 (10)	0.01
**outcome parameters**			
**ventilation, n (%)**	7 (63.6)	0 (0)	<0.001
**seizures, n (%)**	7 (63.6)	1 (10)	0.01
**EEG pathological, n (%)**	10 (90.9)	4 (40)	0.012

**Figure 2:**
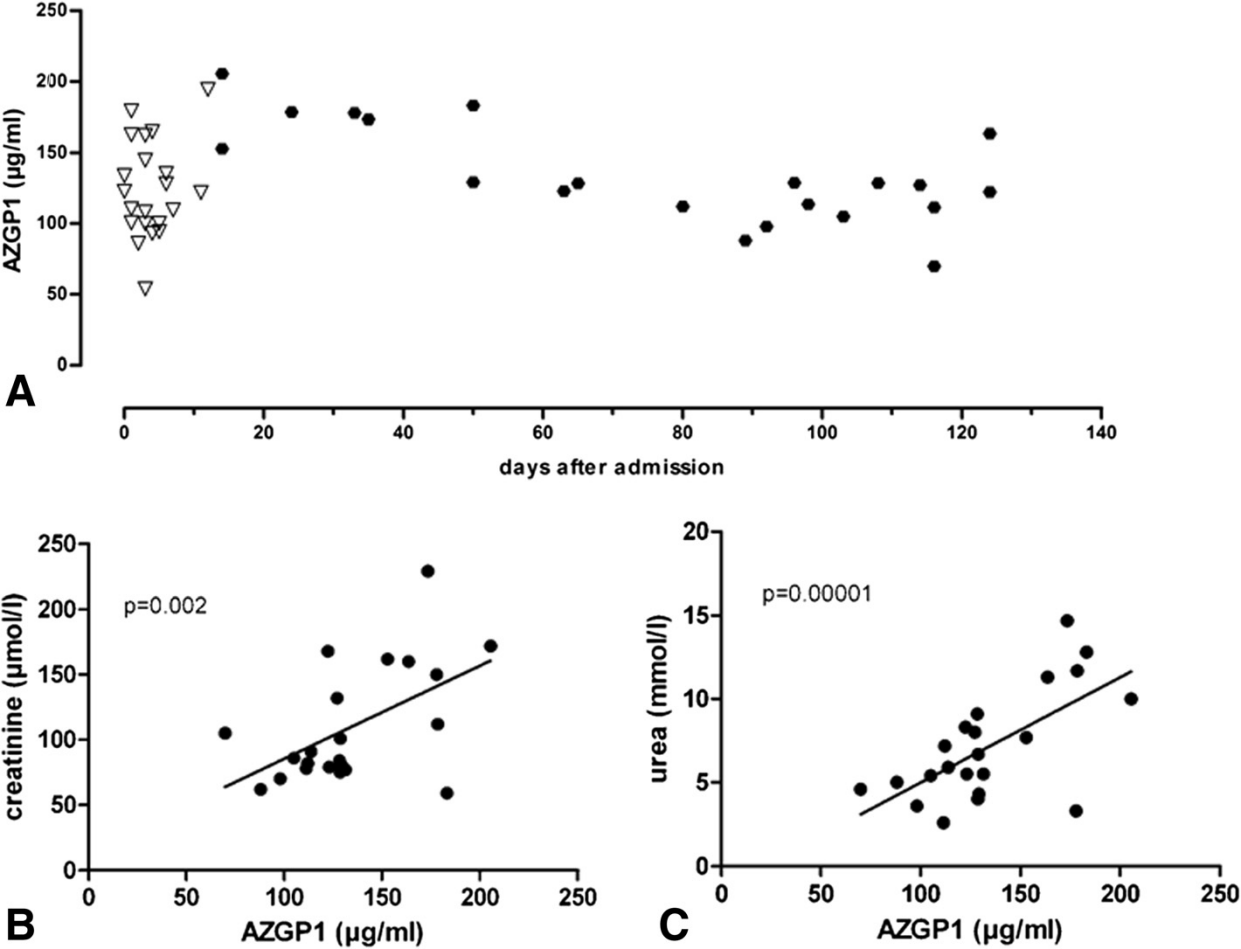
**AZGP1 serum levels correlate with parameters of renal function at follow-up measurements after AKI. A**: AZGP1 serum level s in patients by days after admission to hospital. Initial measurements are depicted as triangles, follow-up measurements as circles. **B**,**C**: Mean AZGP1 levels at follow-up (85 (44 – 100) days after the initial measurement) significantly correlated with creatinine and urea.

Kidney function in all AKI patients recovered until they were discharged after 34 (27 – 38) hospital days, as judged by lack of need for further renal replacement therapy. AZGP1 levels were determined again in a follow-up ambulatory visit at 85 (44 – 100) days after the initial measurement (Figure 2A). At this time 50% of AKI patients showed a further increase in individual AZGP1 levels while 25% had unchanged levels and 25% a decrease in circulating AZGP1. Mean AZGP1 values did not significantly change but we found an inverse correlation between the time after initial measurement and individual follow-up levels (r = −0.6125; p = 0.0032). AZGP1 at follow-up correlated with increased creatinine, urea and eGFR (Figure 2B – C, Table 1). Higher AZGP1 was also associated with increased AST, ALT and LDH (Table 1).

Stepwise linear regression analysis with forward selection revealed serum creatinine (β = .605, p = 0.005) and LDH (β = .185, p = 0.022) as only factors independently associated with AZGP1. Patients presenting serum creatinine levels within normal limits (< 84 μmol/l for females, < 104 μmol/l for males) showed significantly lower AZGP1 values (113.69 (104.65 – 128.42) μg/ml) as compared to patients with incomplete renal recovery (158.17 (127.44 – 176.69) μg/ml; p = 0.012 ).

End stage renal disease has been shown to be associated with increased AZGP1 levels [15,16]. We confirmed this finding in a cohort of 20 chronic HD patients (Figure 1A). In these patients circulating AZGP1 levels where higher than in the AKI cohort. Given the suggested role of AZGP1 in lipid metabolism and body composition we analyzed AZGP1 levels with regard to age, BMI, total body fat as measured by bioelectrical impedance analysis, triglycerides, LDL, HDL, total cholesterol and total protein. In our cohort AZGP1 levels did not associate with any of these parameters (Table 3).

**Table 3:** Univariate correlation between serum AZGP1 levels and other variables in chronic HD patients

	**Chronic HD patients**	
	**n = 20**	
	**Median (quantiles)**	**Pearsons’s coefficient; p-value**
**age** (years)	63 (54–68)	r = −0.2; p = 0.323
**BMI**	25.9 ± 5	r = −0.28; p = 0.23
**total body fat** (%)	34 ± 9	r = −0.07; p = 0.721
**triglycerides** (mg/dl)	194 (122–302)	r = 0.05; p = 0.81
**LDL** (mg/dl)	101 (80–109)	r = −0.003; p = 0.893
**HDL** (mg/dl)	41 (30–48)	r = 0.05; p = 0.81
**total cholesterol** (mg/dl)	178 (148–218)	r = 0.133; p = 0.587
**total protein** (g/l)	6.8 (6.6 - 7)	r = −0.204; p = 0.307

## Discussion

Our study is the first to assess AZGP1 levels in AKI patients. We studied patients of the STEC-HUS cohort who presented with a very rapid and homogenous onset of AKI [18]. We found that the acute rise of serum creatinine in these patients was paralleled by a sharp increase in circulating AZGP1. Patients with stronger elevations of AZGP1 were older and developed significantly more extra-renal complications such as seizures or the requirement of mechanical ventilation. While follow-up AZGP1 levels correlated inversely with parameters of renal recovery they correlated positively with serum markers of cell damage.

An interesting finding of our study was that higher initial levels of AZGP1 correlated with a more severe outcome and with extra-renal complications in STEC-HUS AKI. This increase in circulating AZGP1 might either result from enhanced synthesis and cellular release or it could reflect disrupted elimination. While there is no indication that AZGP1 synthesis is increased during acute illness there is data from almost 40 years ago suggesting that renal function and serum AZGP1 levels are inversely related [21]. Along these lines, it has been noted by Philipp et al. that enhanced AZGP1 levels in CKD patients might be best explained by an accumulation effect due to the loss of normal renal elimination [16]. It has also been suggested that increased levels of AZGP1 in heart failure patients might be due to diminished renal function [12].

With a size of 40 kD AZGP1 is predicted to be normally filtered in the glomerulum and thereafter cleared by the proximal tubule through reabsorption and lysosomal degradation [22]. Accordingly, our results show that the acute elevation in AZGP1 during AKI persisted if renal recovery was incomplete. Highest levels were observed in chronic HD patients pointing to a maximal accumulative effect. In the AKI cohort we observed an association between initial AZGP1 levels and pre-existing comorbidities, such as diabetes, hypertension and coronary heart disease. Although all AKI patients had normal documented prior creatinine values our study can not exclude subclinical renal impairment. The general lack of correlation between renal functional parameters and AZGP1 during early AKI might reflect the fact that this phase of AKI is characterized by rapid dynamic changes and significantly altered kinetics for creatinine and urea that are highly variable until a steady-state is reached.

Adipose tissue is in crosstalk with many organ systems through the secretion of adipokines. A growing number of adipokines, such as leptin and adiponectin, has been identified and a mechanistic link to the pathogenesis of insulin resistance, metabolic syndrome and dyslipidaemia has been established [23,24]. AZGP1 which is also expressed in adipocytes was suggested to act as a novel adipokine [25]. In human visceral and subcutaneous fat AZGP1 expression is negatively associated with increased adiposity and the parameters of insulin resistance [25]. These observations lead to the hypothesis that AZGP1 might be protective in obese individuals exerting anti-obese and anti-diabetic effects [26]. In agreement with two previous reports [15,16], we observed substantially higher levels of AZGP1 in CKD patients. However, we found no correlation between individual AZGP1 levels and body fat, BMI, triglycerides or cholesterol. We can not rule out that a broader analysis including lipid metabolites such as oxidized LDL might have revealed AZGP1 associated changes. An additional limitation of our study is the small sample size which reduced the power to detect more subtle associations.

## Conclusion

In summary, acute and chronic loss of renal function is associated with a significant increase in circulating AZGP1. The most likely reason is an imbalance between endogenous production and physiological elimination of the protein by the kidney. In the STEC-HUS AKI cohort initial AZGP1 levels correlated with the severity of the disease course and extra-renal complications. This might be explained by pre-existing subclinical renal functional impairment in patients who were sicker but could also imply additional pathophysiological effects of AZGP1. In chronic HD patients we found no association of individual AZGP1 levels to parameters of fat metabolism. Further research is needed to explore additional potential effects of increased AZGP1 in patients with kidney disease.

## Competing interests

The authors have no competing interests.

## Authors’ contributions

IS-Z and RS designed the study. IS-Z, JM and JB collected blood samples and performed the AZGP1 measurements. HH, JM, IS-Z, JB, BMWS and RS evaluated the data and wrote the manuscript. All authors read and approved the final manuscript.

## Pre-publication history

The pre-publication history for this paper can be accessed here:

http://www.biomedcentral.com/1471-2369/14/145/prepub
